# Dietary Supplementation with *Camelina sativa* (L. Crantz) Forage in Autochthonous Ionica Goats: Effects on Milk and Caciotta Cheese Chemical, Fatty Acid Composition and Sensory Properties

**DOI:** 10.3390/ani11061589

**Published:** 2021-05-28

**Authors:** Maria Antonietta Colonna, Francesco Giannico, Vincenzo Tufarelli, Vito Laudadio, Maria Selvaggi, Giuseppe De Mastro, Luigi Tedone

**Affiliations:** 1 Department of Agricultural and Environmental Science, University of Bari Aldo Moro, 70126 Bari, Italy; mariaantonietta.colonna@uniba.it (M.A.C.); giuseppe.demastro@uniba.it (G.D.M.); luigi.tedone@uniba.it (L.T.); 2 Department of Veterinary Medicine, University of Bari Aldo Moro, 70010 Bari, Italy; francesco.giannico@uniba.it; 3 Department of DETO, Section of Veterinary Science and Animal Production, University of Bari Aldo Moro, 70010 Bari, Italy; vincenzo.tufarelli@uniba.it (V.T.); vito.laudadio@uniba.it (V.L.)

**Keywords:** *Camelina sativa*, forage, milk quality, fatty acids, Caciotta cheese, Ionica goat

## Abstract

**Simple Summary:**

*Camelina sativa* (L. Cranz), commonly known as false flax, is an annual dicot species belonging to the *Brassicaceae* family. The plant is characterised by extreme rusticity and is able to grow in different climatic and soil conditions, including those of the Southern Italy marginal areas. Camelina is studied as a sustainable crop for food and non-food exploitation. Its interest in animal nutrition is due to the fatty acid (FA) composition of its oil, high in polyunsaturated FA (PUFA). This study aimed to evaluate the effect of dietary supplementation with *Camelina sativa* fresh forage in autochthonous goats on the chemical composition and fatty acid profile of milk and Caciotta cheese, a traditional short-term ripened cheese with a soft ivory-coloured rind, compact texture, semi-soft paste, acid fermented odour, and delicate sweet taste. The milk from the *Camelina* group showed a greater content of conjugated linoleic acid (CLA) and PUFA, with benefits for human health. Under the nutritional point of view, Caciotta cheeses were similar between the two groups, but the recruited panellists appreciated the cheese obtained from the *Camelina* group more for its better palatability.

**Abstract:**

The research studied the effects of dietary supplementation with *Camelina sativa* fresh forage on the chemical and fatty acid composition of milk and Caciotta cheese, and its sensory properties. Twenty Ionica goats were randomly assigned to the following two groups (*n* = 10): the control received a traditional forage mixture (*Avena sativa*, 70%; *Vicia sativa*, 20%; *Trifolium* spp., 10%), while the experimental group was given *Camelina sativa* fresh forage (CAM). All of the dams grazed on pasture and received a commercial feed (500 g/head/day) at housing. The milk from the CAM group showed a higher (*p* < 0.05) content of dry matter, fat, lactose and concentrations of C6:0, C11:0, C14:0, C18:2 *n*-6, CLA and PUFA, while lower (*p* < 0.05) amounts of C12:0, C18:0 and saturated long chain FA (SLCFA). The Caciotta cheese from the CAM group showed a greater (*p* < 0.05) content of *n*-6 FA and *n*-6/*n*-3 ratio, although close to four, thus resulting adequate under the nutritional point of view. The overall liking, odour, taste, hardness, solubility and “goaty” flavour were better (*p* < 0.05) in the CAM cheeses. Further investigation would be advisable in order to evaluate the effect of feeding *Camelina* forage obtained from different phenological stages, and the application of ensiling techniques.

## 1. Introduction

*Camelina sativa* (L. Crantz), commonly known as false flax, is an annual dicot species belonging to the Brassicaceae family, it originated in Asia and was introduced in Europe around the 16th century [1,2]. Both winter and spring biotypes are available, and within each biotype, different cultivars, genotypes and GenBank accessions are present [3]. The plant is characterised by extreme rusticity and flexibility, and is able to grow with success in different climatic and soil conditions, including those of the marginal areas [3,4,5]. *Camelina sativa* is naturally resistant to several diseases and insect pests; therefore, the cultivation of the crop is simple and environmentally friendly, since the use of pesticides/herbicides is unnecessary [6,7]. The potential of *Camelina sativa* as a low-input crop for food (cookies, energy drinks, yogurt, salad dressings, cereal and granola bars, etc.) and non-food exploitation (animal feeding, cosmetics, soil fungicides, bio-oils, bio-fuel) has been studied over the last three decades [8,9,10].

The constant interest over time towards *Camelina sativa* is principally related to the fatty acid (FA) composition of its oil, which is exceptionally high in polyunsaturated FA (PUFA > 50% of total FA) [11], as well as to the presence of health-beneficial compounds such as phenols, flavonoids, and molecules with a strong radical-scavenging activity [12]. 

Although *Camelina* has been used as seed, oil expeller cake and meal in the diet of laying hens [13], broilers [14], pigs [15], rabbits [16] and lambs [17], it contains also some antinutritive factors, such as glucosinolates, sinapines, phytic acid, trypsin inhibitors and tannins [18,19]. Czerniawski et al. [20] found that anti-nutritional compounds are principally present in the seeds and roots of *Camelina*. Moreover, previous studies reported that the levels of sinapine, phytic acid, and condensed tannins in *Camelina sativa* meal are lower as compared to the other Brassicaceae species generally used in animal feed formulations [21]. The most important antinutritive components in Camelina meal are glucosinolates, but it should be emphasized that ruminants are more tolerant to glucosinolates than other species. Although Camelina is also characterized by the presence of phytic acid, this is a major concern when it is fed to monogastric animals because, unlike ruminants, they lack the enzymes able to hydrolyze the phosphorus bound [21]. 

As for milk and dairy production, Hurtaud and Peyraud [22] found that dietary inclusion of *Camelina sativa* seeds or meal affected the cow’s milk fat content and the butter’s firmness and spreadability. Pikul et al. [23] found that milk and kefir obtained from goats fed with *Camelina sativa* cake were characterized by increased levels of beneficial nutritional factors, including monounsaturated FA (MUFA) and PUFA, in turn of a decrease in the total saturated FA (SFA). In ewes, dietary supplementation with *Camelina sativa* cake reduced the level of short-, medium- and long-chain saturated FA in milk, with a simultaneous increase in the content of unsaturated FA and their conjugated isomers [24].

Many studies have reported that grazing on pasture and feeding fresh forages are able to affect the FA profile in ruminant milk by enhancing the concentration of conjugated linoleic acid (CLA), α-linolenic, and rumenic and vaccenic acids [25,26]. Modifications in milk fatty acid composition through nutrition might result in positive or adverse changes in the nutritional properties of goat cheeses as well as for its sensorial attributes [27,28].

Caciotta is a fresh or semi-seasoned soft cheese made using cow, sheep and goat milks, alone or mixed together in different percentages, in relation to the local tradition of cheese-making in the Italian regions [29,30]. The modern consumer is rediscovering traditional dairy products endowed with special sensorial attributes that recall the scents of the natural pastures where the animals graze [31]. The nutritional and health properties of sheep and goat products obtained by environmentally friendly production systems, based on the use of local feed resources, is contributing to the economic revival of small ruminant rearing [31,32,33,34,35,36,37]. Among the autochthonous goat breeds reared in the marginal areas of the Apulia and Basilicata regions, the Ionica goat is well adapted to graze on arid soils, being able to use the poor pasture resources available. Obtained by crossing the autochthonous animals with individuals belonging to the Maltese breed and subsequent crossbreeding, this genotype was widespread in the second half of the 20th century, while nowadays it is listed among the Italian endangered breeds as drawn up by the Department for Environment, Food, and Rural Affairs [38]. Therefore, the optimization of the typical and traditional productions may be an effective way to promote the rescue of this breed.

So far, while *Camelina sativa* has been thoroughly investigated as meal, oil and expeller cake, little information is available on its use as fresh forage in small ruminant feeding.

The aim of the research was to study the effect of dietary supplementation with *Camelina sativa* (L. Crantz) fresh forage in Ionica goats on the chemical composition and fatty acid profile of milk and Caciotta cheese, and on its sensory properties.

## 2. Materials and Methods

### 2.1. Camelina Sativa Forage Production and Analysis

The cultivation of *Camelina sativa* (L. Crantz) was carried out in a farm located in Gravina in Puglia (Apulia, Southern Italy, 40°59′42.22″ N, 16°20′38.82″ E, 345 m above the sea level) as a main crop in winter–spring sowing with an extensive, environmentally friendly and low energy input cultivation technique described elsewhere [39]. Due to its high rusticity, *Camelina sativa* showed a good adaptation to the soil and climatic conditions, with productions of approximately 15 tons/ha of fresh forage. *Camelina sativa* green biomass was harvested daily (about 60 kg by the time) and administered as fresh forage to the experimental goats.

The potential nutritive value and in vitro gas production (IVGP) parameters of *Camelina* were assessed according to the method of Menke et al. [40] as described in Getachew et al. [41]. In vitro incubation was performed using 40 mL of buffered rumen fluid. Approximately 200 mg of ground Camelina was incubated alone in 100 mL graduated glass syringes. Buffered rumen fluid (40 mL) was pipetted into each syringe containing ground Camelina as the substrate. The syringes were immediately placed in a water bath at 39 °C. Each incubation was performed in quadruplicate. Gas production was recorded up to 72 h and the syringes were shaken every hour for the first 8 h of incubation. The total in vitro gas produced was corrected for blank incubations (i.e., no substrate). 

The gas production was expressed in mL/g Dry Matter (DM), while the metabolizable energy (MJ/kg DM) was calculated using the following formula: ME = 1.06 + 0.157GP + 0.084CP + 0.22CF − 0.081A, in which GP is the net gas production in the 72 h (mL/g DM) and CP, CF and A are the values of crude protein, crude fat and ash (%DM), respectively.

### 2.2. Animals, Diet, Management, Milk Sampling and Cheese Making

The trial was carried out during March–May 2017 in an agro-zootechnical farm located in Laterza (province of Taranto, Apulia, Southern Italy; 40°37′49″ N; 16°48′1″ E, 360 m above the sea level). Twenty female pluriparous goats of the autochthonous Ionica breed, homogeneous for weight, parity (3–4), time of kidding (30 ± 5 days after delivery) and milk yield, were randomly assigned to the following two groups (*n* = 10) that differed between each other only for the type of forage administered ad libitum: the control group (C) received a traditional forage mixture (*Avena sativa*, 70%; *Vicia sativa*, 20%; *Trifolium* spp., 10%), while the experimental group (CAM) was given *Camelina sativa* fresh forage. According to the traditional goat rearing system, during the day all the dams grazed on a spontaneous vegetation typical of the Mediterranean area; at housing, in the evening, the dams of the two groups were administered the same commercial feed (500 g/head/day) containing the following (as fed basis): dehydrated alfalfa (18% Crude Protein, CP; 30%), corn (19.5%), barley (20.0%), soybean meal (44% CP; 16%), dried beet pulp (2.9%), soft wheat bran (3%), brewer’s yeast (2%), mineral and vitamin supplements (0.1%), calcium carbonate (0.8%), dicalcium phosphate (0.9%), sodium bicarbonate (0.6%), sodium chloride (0.6%), magnesium oxide (0.6%) and soybean oil (3%). Moreover, at this time they received the forage, which differed between the two experimental groups as described above. 

The experimental period lasted 7 weeks, among which the first 2 weeks were for diet adaptation and the last 5 weeks for milk collection and cheese-making. The chemical composition and fatty acid profile of pasture, hay, pelleted feed and *Camelina sativa* forage is shown in Table 1.

Individual milk samples were collected twice a day (7:00 a.m. and 6:00 p.m.), mixed together and stored at +4 °C. The milk collected per dam was divided into two aliquots, one of which was analyzed for fat, protein and lactose by an infrared milk analyzer (Milkoscan 133-B, Foss Electric, Hillerod, Denmark) previously standardized for goat milk, while the second aliquot was stored at −80 °C until fatty acid analysis was performed. Somatic cells count (SCC) was also assessed and the result (10^3^/mL) was transformed into logarithmic form (log_10_).

For each treatment group, cumulative milk samples were collected daily and processed for Caciotta cheese-making. The raw whole milk was filtered and heated in a stainless vat to 36 °C; liquid calf rennet was added (0.36 mL/L of milk). After 20–25 min, at the end of the coagulation process, the curd was cut with a knife into equal-sized pieces of 10 cm; after 5 min of rest, the curd was broken into the size of rice grains and hand-worked until it reached the characteristic spherical shape. The curd was placed into plastic cylindrical molds of 15 cm in diameter and 11 cm high. After dry salting, the cheeses underwent ripening for 20 days at 85% relative humidity and temperature +10 °C. Each Caciotta was split into two halves, one of which was frozen (−80 °C) in order to carry out chemical and fatty acid analyses, while the other was vacuum packed and refrigerated at +1 °C until sensory analysis was performed. Ten cheeses were analyzed for each treatment group.

### 2.3. Chemical Composition of Feeds, Milk and Cheese

Pasture samples were collected along transects according to the methods previously described [31]. Samples of grass and *Camelina sativa* green biomass were dried at 60 °C for 48 h in a stove, homogenized and analyzed. Samples of the pelleted feed, dried pasture, hay and *Camelina sativa* forage were ground in a hammer mill through a 1-mm sieve and analyzed using the following AOAC international procedures [42]: DM (method 934.01), fat (method 920.39), ash (method 942.05), CP (method 954.01), crude fiber (method 945.18), ADF and ADL (method 973.18), and NDF (method 2002.04). 

Cheese samples were homogenized and lyophilized. Fat content was determined by the Soxhlet method and expressed as fat in DM. Total nitrogen was determined by the Kjeldahl method [42] and expressed as protein content (nitrogen content × 6.38). In milk and cheese samples, ash content was detected after burning a lyophilized sample in a muffle furnace at 550 °C for 5 h.

### 2.4. Fatty Acid Profiles of Feeds, Milk, and Cheese

Total lipids were extracted from the homogenized samples (100 g) according to the chloroform and methanol method described by Folch et al. [43]. Fatty acids (FA) were methylated by using a BF_3_-methanol solution (12% *v/v*) [44]. The FA profile was assessed with a Chrompack CP 9000 gas chromatograph with a silicate glass capillary column (70% cyanopropyl polysilphenylene-siloxane BPX 70 of SGE Analytical Science, Chebios S.r.l., Rome, Italy; length: 50 m; internal diameter: 0.22 mm; film thickness: 0.25 μm). The temperature program was 135 °C for 7 min, followed by increases of 4 °C per minute up to 210 °C. Fatty acid peaks were identified by using a comparative analysis with standard reference mixtures. For the identification of C18:2 cis9, trans11 isomer (conjugated linoleic acid, CLA), a mixture of CLA methyl esters was used (Sigma-Aldrich, Darmstadt, Germany).

For cheese, FAME (fatty acid methyl esters) were prepared by direct transesterification (IUPAC, 1987, method 2.301). Fatty acid analysis was performed according to the procedure described by Caponio et al. [45] using a capillary gas chromatograph (Fison high-resolution gas chromatography, HRGC Mega 2 series; Milan, Italy) equipped with a flame ionization detector fitted with wall-coated open tubular fused-silica capillary column (FFAP-CB coating, 25 m × 0.32 mm i.d. × 0.30 μm film thickness; Chrompack, Middleburg, the Netherlands). The separation was performed at pre-programmed temperatures as follows: 50 °C for 3 min; 50 to 100 °C at a rate of 20 °C/min; 100 °C for 2 min; 100 to 240 °C at a rate of 20 °C/min; 240 °C for 15 min. Hydrogen was the carrier gas (flow rate, 2 mL/min). The injector temperature was 270 °C (splitting ratio, 1:17), and the detector temperature was 300 °C. Fatty acids were expressed as the percentage (wt./wt.) of total FAME.

The following lipid quality indices were calculated on goat milk and Caciotta cheese: the atherogenic (AI) and thrombogenic (TI) indices [46]; the concentration of hypocholesterolemic (DFA) and hypercholesterolemic fatty acids (OFA) [47]; the ratio of hypocholesterolemic and hypercholesterolemic fatty acids (H/H) [48].

### 2.5. Assessment of Caciotta Cheese Colour and Texture Profile Analysis

The colorimetric features (L* = lightness, a* = redness, b* = yellowness) of Caciotta cheese were determined using a HunterLab Miniscan™ XE spectrophotometer (model 4500/L, 45/0 LAV, 3.20 cm diameter aperture, 10° standard observer, focusing at 25 mm, illuminant D65/10; Hunter Associates Laboratory Inc., Reston, VA, USA) by taking three readings on the rind of each cheese sample. The instrument was normalized to a standard white tile provided with the instrument before performing analysis (Y = 92.8, x = 0.3162 and y = 0.3322) [49]. Moreover, the total color difference (ΔE*ab) was also calculated according to the following formula: ∆E*ab = [(∆L*)^2^ + (∆a*)^2^ + (∆b*)^2^]^1/2^ [50].

Evaluation of the rheological properties of Caciotta cheese was performed using an Instron 5544 universal testing machine (Instron Corp., Canton, MA, USA) equipped with a flat steel probe of 25-mm diameter, which simulates the conditions applied during mastication through a double compression test (texture profile analysis) elaborated by the incorporated software. The following experimental conditions were adopted: pre-load = 0.05 N; test speed = 1 mm/s; deformation = 50%). For each experimental group, cheese cubes of 1 cm side, kept at 20 °C for 30 min, were evaluated in triplicate. Deformation at the first bite (mm), springiness (mm), gumminess (N) and chewiness (N × mm) were evaluated.

### 2.6. Panel Test

The sensory properties of cheese were evaluated by a group of 10 panelists that had prior experience with descriptive analysis (5 males and 5 females, 25–45 years old) at the University of Bari campus. A small amount (20 g) of cheese from both C and CAM groups, for a total of 20 samples (no. = 10/group), was placed in coded white small plastic plates in a randomized order. All the samples were left at room temperature (about +20 °C) for 30 min before administration [51] and were served together with non-salted crackers and still water. For visual evaluation, the judge looked at the uniformity of rind and paste colors (0 = not uniform; 9 = very uniform) and of the internal structure (0 = uniform; 9 = presence of eyes). As for the evaluation of the aroma, the judge evaluated the intensity of the odor (0 = blank; 9 = very intense) regarding the following descriptors: cabbage, turnip, herbaceous, mold, oil, butter and goat. The judges evaluated, also on a 9-point scale, the flavor (salty, sweet, acid) and the texture (hardness, adhesiveness and solubility following mastication). After tasting, each panelist rated the overall liking of the product (0 = strongly disliked; 9 = strongly liked). 

### 2.7. Statistical Analysis

All the data, repeated three times for each treatment, were subjected to analysis of variance (ANOVA). Means were separated and compared by Tukey’s Honestly Significant Difference (HSD) test.

## 3. Results and Discussion

### 3.1. Chemical and Fatty Acid Composition of Goat Milk

The chemical and fatty acid composition of milk obtained from grazing goats, fed a control pelleted diet in association with hay or with *Camelina sativa* forage, are presented in Table 2. The milk from the Camelina group showed a significantly (*p* < 0.05) higher content of dry matter, fat and lactose in comparison with the control one. In the present study, the chemical composition of the milk from the Ionica goats is comparable to that reported by Di Trana et al. [30], who carried out a comparative study on milk chemical and fatty acid composition in four goat breeds commonly reared in South Italy regions. Our findings, in terms of milk chemical composition, fall within the range values recorded by Rolinec et al. [52] in goats raised in conditions very close to ours, i.e., grazing on pasture and supplemented with a commercial pelleted feed at housing.

The content of the somatic cells in the goat milk did not differ between the groups, and it was lower in comparison with the results recorded by Kováčová et al. [53] during April–May, which is the same period in which the present experiment was conducted. The SCC score is used for the evaluation of milk quality; it is an indicator of the mammary gland health and hygiene, and it varies widely in relation to breed, month of milking and farming conditions [54], taking into consideration that goats are generally hand-milked in small family processing plants following traditional procedures [55].

As for the FA profile of goat milk, supplementation with *Camelina* forage significantly (*p* < 0.05) increased some saturated FA, such as C6:0, C11:0 and C14:0, while it lowered (*p* < 0.05) the concentration of C12:0 and C18:0. Lauric (C12:0), myristic (C14:0) and palmitic (C16:0) acids are the SFA able to increase blood cholesterol levels with negative consequences on the cardiovascular system; therefore, a decrease in their concentration in animal products is a desirable goal [56,57]. Since C18:0 is the most representative FA among SLCFA, this class of FA was more abundant in milk from the control group as compared with the *Camelina* one. The distribution of short-, medium- and long-chain FA is quite different from that reported by Cossignani et al. [58], especially with regards to the amount of SMCFA and SLCFA, which were 27.7% and 35.8%, respectively, in comparison with the average of 46.8% and 8.5% found in our study. The differences in the FA profile of milk may be attributable to the goat genotype, stage of lactation, feeding regimen or to individual factors [52,53,54]. 

Supplementation with *Camelina sativa* forage significantly (*p* < 0.05) enhanced the concentration of linoleic acid (C18:2, *n*-6), CLA, and PUFA, with benefits for human health [59]. Pikul et al. [23] found an almost two-fold greater concentration of CLA and a higher content of unsaturated FA, both MUFA and PUFA, in milk from goats supplemented with false flax cake. Ruminant dairy products are known to be the major dietary source of CLA, in particular of rumenic acid, which is the cis-9, trans-11 isomer, whose concentration increases in milk from grazing animals [60,61]. CLA has a well-documented effect in preventing atherosclerosis, different types of cancer and hypertension, besides exerting positive effects on the immune response [56,57,58,59]. The milk average CLA concentration found in this trial is higher in comparison with the results reported in a previous study carried out on commercial goat milk [58]. The concentration of total fatty acids of the *n*-6 series was significantly increased (*p* < 0.05) following supplementation with *Camelina* forage and, as a consequence, also the *n*-6/*n*-3 ratio was greater (*p* < 0.05). According to Wood et al. [62], the optimal value of the *n*-6/*n*-3 ratio should be 4:1 or less. In this study, the *n*-6/*n*-3 ratio found for both of the groups is suitable and similar to the findings reported by other authors [58,61]. No differences between dietary treatments arose for any of the lipid quality indices assessed in goat milk. The atherogenic index found in milk from both of the groups falls within the range of 2.20–3.29, which is in agreement with the results reported in the literature for goats’ milk [47]. 

### 3.2. Chemical and Fatty Acid Composition of Goat Caciotta Cheese

Table 3 shows the results of the chemical and fatty acid profile of goat Caciotta cheese. The dietary treatment did not affect the chemical composition of the cheeses, in accordance with the findings reported in a study conducted on kefir produced from goats fed a false flax supplement [23]. The *Camelina* diet significantly lowered (*p* < 0.05) the concentration of stearic acid (C18:0), while it determined an increase (*p* < 0.05) in the content of arachidonic (C20:4 *n*-6) acid. Goat milk and cheeses contain more essential FA, such as linoleic and arachidonic acids, compared to cow dairy products [63]. In ruminant milk, linoleic acid is required for the synthesis of arachidonic acid, which is the precursor of eicosanoids, including prostaglandins, thromboxanes, and leukotrienes. These have key roles in the regulation of inflammation, immunity, platelet aggregation, smooth muscle contraction and kidney function. Inappropriate or excess production of these eicosanoids leads to disease occurrence [64]. 

Similarly to the results obtained for goat milk, also Caciotta cheese produced from the Camelina group showed a significantly higher (*p* < 0.05) content of FA of the *n*-6 series, as well as a greater *n*-6/*n*-3 ratio. However, for both of the groups the value of the *n*-6/*n*-3 ratio was close to four, thus resulting as adequate from the nutritional point of view.

Accordingly to the findings recorded for goat milk, the lipid quality indices of the Caciotta cheeses did not statistically differ between the groups. A comparative study conducted on cow, sheep and goat hard cheeses put in evidence similar values of H/H between the three species. However, the upper limit found for the mean range of goat cheese was slightly higher (0.75) in comparison with the values of 0.60 and 0.63 found for cow and sheep cheeses, respectively. The H/H ratio is related to the metabolism of lipoproteins involved in plasma cholesterol transport, and to the risk of cardiovascular disease development. Higher values of this ratio, as obtained in our study, have been reported to be desirable [65,66].

### 3.3. Assessment of Caciotta Cheese Colour and Texture Profile Analysis

Table 4 shows the results of the instrumental analysis performed on goat Caciotta in order to assess the cheese color features and the rheological properties following compression. No statistical difference arose between the two dietary treatments, neither for the color nor for the texture profile. 

The L* value of the Caciotta cheeses found in the present study is slightly higher in comparison with the findings reported by Innosa et al. [67] on Caciotta cheeses obtained from a different goat breed and ripened for a longer period, but lower in comparison with the results reported by Kováčová et al. [53] for cheeses ripened only for 12 days. The duration of the ripening period, by affecting the moisture content and the occurrence of oxidative processes involving fats and proteins, seems to be the major factor able to influence the lightness in a dairy product [67]. The higher b* value found for the cheese of the Camelina group, although not statistically significant (*p* = 0.06), may be due to the higher content of fat-soluble vitamins in the Camelina fresh forage and in its flowers, responsible of the tendency for the cheese to take on a yellowish color. Ruminant diets containing large amounts of green forage determine an increase in the concentration of the pigments in milk, among which the most common is carotene, which contributes to the yellow coloration of dairy products [68]. The ∆E*ab calculated in the present study, in order to compare the overall color differences between the cheeses from the two treatment groups, was equal to 0.96; according to the criteria suggested by Larraín et al. [50], changes in the instrumental color measurements may be visually noticeable when the ΔE*ab value falls within a range between 2 and 6. Based on this statement, in our trial, the ΔE*ab value of 0.96 is too low to be visible to the average person. As a matter of fact, this evidence is confirmed by the results of the panel test described onwards.

The diet did not affect any of the rheological characteristics of the Caciotta cheese, even though slightly lower values of gumminess and chewiness were recorded following the Camelina diet, which may be related to the higher concentration of fatty acids of the *n*-6 series found in the cheeses of this group. Similarly, Hurtaud and Peyraud [22] found that *Camelina*-based diets provided butters that were less firm and spreadable, as a consequence of the greater concentration of unsaturated fatty acids. 

### 3.4. Sensory Properties of Caciotta Cheese

Table 5 shows the results of the panel test carried out on the Caciotta cheeses. The overall liking of Caciotta cheese from the Camelina-based diet was significantly (*p* < 0.05) higher in comparison with the control group. Furthermore, the panelists gave better scores (*p* < 0.05) to the Caciotta cheese from the Camelina group also in terms of odor, taste and consistency, since the improved hardness and solubility features were recorded. The Camelina-based diet determined a significantly (*p* < 0.05) higher “goaty” flavor in comparison with the Caciotta cheeses obtained from the control group, which is a desirable sensory attribute. The other descriptors used to judge the Caciotta cheese were quite similar between the two groups, thus the general opinion expressed by the trained panelists was fully acceptable with regards to the cheese obtained from the Camelina-fed goats. Pikul et al. [23] reported no statistically significant effect on the organoleptic features of kefir produced from goats supplemented with false flax cake. There is limited information in research papers focusing on the effect of feeding with *Camelina sativa* as forage on the fatty acid profile of milk and cheeses in small ruminants. Moreover, the few data available are hardly comparable since there are many different variables. Goat Caciotta cheeses may differ greatly depending on the characteristics of the goat milk as well as on the cheese-making technique [30,31]. Furthermore, the duration of cheese ripening is a very important feature that affects the sensory properties of cheese, since flavor compounds develop as a result of the activity of cheese bacteria [55], and of the metabolic pathways involving fat, lactose and proteins [69].

## 4. Conclusions

This is a preliminary study aiming to evaluate the possibility to use the whole plant of *Camelina sativa* (L. Crantz) during the flowering stage of growth, as fresh forage for autochthonous goats reared by an extensive and environmentally friendly system, on the fatty acid profile of milk and Caciotta cheese. Although dietary supplementation with *Camelina* fresh forage determined an increase in the concentration of myristic acid in milk, in turn it enhanced the content of CLA and PUFA, with potential benefits for human health.

From the nutritional point of view, the Caciotta cheeses were similar between the two groups. The recruited panelists appreciated more the cheese obtained from goats fed with *Camelina* forage, which was judged to have a satisfactory palatability. Further investigation would be advisable to evaluate the effect of feeding *Camelina sativa* forage obtained from different phenological stages of the development of the plant, along with the application of ensiling techniques in order to enable the forage preservation. 

## Figures and Tables

**Table 1:** Chemical composition (%DM) and fatty acid profile (%FAME) of pasture, hay, pelleted feed and *Camelina sativa* forage.

	Pasture	Hay	Pelleted Feed	*Camelina sativa* Forage
Chemical composition				
Dry matter (%)	25.80	92.30	92.50	19.40
Crude Protein	18.76	13.54	19.03	11.34
Ether extract	5.03	2.80	3.49	6.20
Ash	9.78	10.51	7.61	10.82
Crude fiber	27.51	40.78	9.18	21.65
N-free extracts	38.92	32.37	60.69	49.99
NDF	57.65	63.58	38.82	49.50
ADF	34.50	35.08	11.17	24.70
ADL	6.31	11.25	4.61	4.65
AIA	1.87	0.92	0.37	3.70
IVGP (mL/g DM)	195.50	256.10	288.60	180.77
ME (MJ/kg DM)	9.30	11.40	13.20	7.27
Fatty acids				
C_14:0 (Myristic)_	0.00	0.61	0.30	0.63
C_16:0 (Palmitic)_	21.20	13.56	13.50	18.59
C_18:0 (Stearic)_	0.20	1.60	3.20	0.10
C_18:1n9cis (Oleic)_	25.20	14.60	25.10	7.90
C_18:2n6__(Linoleic)_	13.30	31.60	50.10	13.50
C_18:3n3 (α-linolenic)_	12.20	6.50	3.20	43.25
C_20:0 (Arachidic)_	0.10	0.60	0.30	3.23
C_20:1 (Gondoic)_	0.10	0.10	-	0.20
C_22:0 (Behenic)_	-	-	-	0.75
C_22:1 (Erucic)_	-	-	-	-

DM: Dry Matter; FAME: Fatty acid methyl esters; NDF: Neutral Detergent Fiber; ADF: Acid Detergent Fiber; ADL: Acid Detergent Lignin; AIA: Acid Insoluble Ash; IVGP: In Vitro Gas Production; Metabolizable Energy.

**Table 2:** Chemical and fatty acid composition of goat milk (mean ± SEM).

Variable	Treatment		SEM ^1^
	Control	Camelina	
Chemical composition (% DM)			
Dry Matter	12.96 ^b^	13.47 ^a^	0.41
Fat	4.11 ^b^	4.33 ^a^	0.14
Protein	3.61	3.72	0.14
Ash	0.70	0.80	0.09
Lactose	4.54 ^b^	4.62 ^a^	0.05
Somatic cells (mean log_10_)	3.19	3.24	0.10
Fatty acids (% FAME)			
C4:0	1.25	1.28	0.11
C6:0	1.83 ^b^	2.76 ^a^	0.25
C8:0	3.35	2.91	0.32
C10:0	11.30	10.32	0.93
C11:0	0.06 ^b^	0.16 ^a^	0.02
C12:0	6.13 ^a^	5.20 ^b^	0.49
C13:0	0.15	0.18	0.02
C14:0	6.31 ^b^	10.89 ^a^	1.00
C14:1	0.40	0.56	0.09
C15:0	1.45	1.55	0.14
C16:0	19.41	18.11	0.60
C16:1	0.63	0.68	0.08
C17:0	1.20	1.23	0.13
C18:0	9.48 ^a^	6.48 ^b^	0.72
C18:1 *n*-9	15.23	14.97	0.95
C18:2 *n*-6	2.47 ^b^	3.30 ^a^	0.19
CLA, c9t11	0.71 ^b^	0.91 ^a^	0.08
C18:3 *n*-3	0.40	0.44	0.04
C20:0	0.49	0.48	0.04
C20:4 *n*-6	0.23	0.21	0.04
C20:5 *n*-3	0.29	0.22	0.02
C22:6 *n*-3	0.09	0.06	0.01
Total SFA	62.41	61.55	3.09
Total MUFA	16.26	16.29	1.02
Total PUFA	4.34 ^b^	5.35 ^a^	0.22
Other acids	16.99	16.81	1.28
SSCFA (Saturated Short Chain FA)	6.43	6.95	0.48
SMCFA (Saturated Medium Chain FA)	46.01	47.64	2.30
SLCFA (Saturated Long Chain FA)	9.97 ^a^	6.96 ^b^	0.74
Total *n*-6	3.40 ^b^	4.42 ^a^	0.20
Total *n*-3	0.94	0.93	0.07
*n*-6/*n*-3	3.62 ^b^	4.75 ^a^	0.47
AI (Atherogenic Index)	2.65	3.08	1.07
TI (Thrombogenic Index)	2.93	2.66	0.67
DFA (Hypocholesterolemic FA)	30.07	28.03	2.08
OFA (Hypercholesterolemic FA)	31.85	34.20	2.09
H/H (DFA/OFA)	0.95	0.82	0.16

^1^ Standard error of means. Differences between diets: ^a,b^: *p* < 0.05. DM: Dry Matter; FAME: Fatty acid methyl esters; SFA: Saturated Fatty Acids; MUFA: Mono Unsaturated Fatty Acids; PUFA: Poly Unsaturated Fatty Acids; SSCFA: saturated fatty acids from C4:0 to C8:0; SMCFA: saturated fatty acids from C10:0 to C17:0; SLCFA: saturated fatty acids from C18:0 to C24:0.

**Table 3:** Chemical and fatty acid composition of goat Caciotta cheese (mean ± SEM).

Variable	Treatment		SEM ^1^
	Control	Camelina	
Chemical composition (% DM)			
Dry Matter (%)	54.31	53.67	0.53
Fat	15.76	15.52	0.46
Protein	26.22	25.78	0.61
Ash	2.70	2.61	0.11
N-free extracts	9.63	9.76	0.23
Fatty acids (% FAME)			
C4:0	1.18	1.20	0.09
C6:0	1.84	2.99	0.37
C8:0	3.78	3.59	0.29
C10:0	13.48	12.06	0.56
C11:0	0.11	0.16	0.02
C12:0	5.32	5.27	0.44
C13:0	0.16	0.17	0.01
C14:0	8.56	10.30	0.53
C14:1	0.37	0.22	0.02
C15:0	1.68	1.67	0.18
C16:0	20.10	18.32	0.69
C16:1	0.87	1.27	0.12
C17:0	1.55	1.11	0.19
C18:0	8.82 ^a^	7.16 ^b^	0.72
C18:1 *n*-9	17.51	17.19	0.43
C18:2 *n*-6	2.86	3.20	0.10
CLA, c9t11	0.86	0.83	0.10
C18:3 *n*-3	0.51	0.47	0.06
C20:0	0.58	0.49	0.07
C20:4 *n*-6	0.04 ^b^	0.30 ^a^	0.06
C20:5 *n*-3	0.08	0.10	0.04
C22:6 *n*-3	0.32	0.24	0.01
Total SFA	67.16	64.49	1.97
Total MUFA	18.43	17.20	0.53
Total PUFA	4.87	5.38	0.14
Other acids	9.54	12.93	1.97
SSCFA	6.80	7.78	1.36
SMCFA	50.96	49.06	1.48
SLCFA	9.40	7.65	1.96
Total *n*-6	3.76 ^b^	4.33 ^a^	0.32
Total *n*-3	1.11 ^a^	1.05 ^b^	0.27
*n*-6/*n*-3	3.38 ^b^	4.12 ^a^	1.30
Index of atherogenicity (IT)	2.59	2.64	0.11
Index of thrombogenicity (IA)	2.59	2.36	0.12
DFA (Hypocholesterolemic FA)	32.11	31.67	1.14
OFA (Hypercholesterolemic FA)	34.04	33.89	0.86
H/H (DFA/OFA)	0.94	0.93	0.05

^1^ Standard error of means. Differences between diets: ^a,b^: *p* < 0.05. DM: Dry Matter; FAME: Fatty acid methyl esters; SFA: Saturated Fatty Acids; MUFA: Mono Unsaturated Fatty Acids; PUFA: Poly Unsaturated Fatty Acids; SSCFA: saturated fatty acids from C4:0 to C8:0; SMCFA: saturated fatty acids from C10:0 to C17:0; SLCFA: saturated fatty acids from C18:0 to C24:0.

**Table 4:** Color evaluation and texture profile analysis of goat Caciotta cheese (mean ± SD).

Descriptor	Treatment	
	Control	Camelina
L*	60.87 ± 2.32	60.12 ± 2.10
a*	−2.50 ± 0.32	−2.61 ± 0.27
b*	2.25 ± 0.19	2.84 ± 0.23
Deformation (mm)	6.30 ± 1.41	6.42 ± 1.18
Springiness (mm)	8.90 ± 1.10	8.83 ± 0.98
Gumminess (N)	21.10 ± 1.01	20.80 ± 1.33
Chewiness (N × mm)	120.30 ± 10.64	115.70 ± 11.95

SD: Standard deviation; L*: lightness, a*: redness, b*: yellowness.

**Table 5:** Sensory properties of goat Caciotta cheese (mean ± SD).

Descriptor	Treatment	
	Control	Camelina
Cabbage	0.00 ± 0.32	0.10 ± 0.01
Turnip	0.20 ± 0.32	0.10 ± 0.04
Herbaceous	0.90 ± 0.79	0.80 ± 0.10
Mold	1.40 ± 1.10	1.10 ± 0.9
Oil	3.30 ± 1.41	3.30 ± 1.18
Butter	0.90 ± 0.80	0.90 ± 0.80
Goat	2.10 ± 1.01 ^b^	3.80 ± 2.33 ^a^
Sweet	3.30 ± 1.64	2.70 ± 1.95
Salty	5.90 ± 2.50	5.70 ± 3.60
Acid	3.70 ± 2.07	3.50 ± 1.80
Hardness	5.50 ± 2.05 ^b^	7.00 ± 2.50 ^a^
Adhesiveness	1.88 ± 1.66	2.10 ± 0.80
Solubility	4.50 ± 2.50 ^b^	5.20 ± 2.99 ^a^
Colour	8.00 ± 0.67	8.00 ± 0.60
Structure	1.30 ± 0.67	1.30 ± 0.60
Odour	6.50 ± 0.67 ^b^	7.60 ± 0.50 ^a^
Taste	6.50 ± 0.32 ^b^	7.70 ± 0.80 ^a^
Overall liking	6.50 ± 0.72 ^b^	7.80 ± 0.50 ^a^

SD: Standard deviation. Differences between diets: ^a,b^: *p* < 0.05.

## Data Availability

The datasets used and/or analyzed during the current study are available from the first author and from the corresponding author on reasonable request.

## References

[1] Zubr J. (1997). Oil-seed crop: Camelina sativa. Ind. Crops Prod..

[2] Jankowski K.J., Sokólski M. (2021). Spring camelina: Effect of mineral fertilization on the energy efficiency of biomass production. Energy.

[3] Zanetti F., Alberghini B., Jeromela A.M., Grahovac N., Rajković D., Kiprovski B., Monti A. (2021). Camelina, an ancient oilseed crop actively contributing to the rural renaissance in Europe. A review. Agron. Sustain. Dev..

[4] Martinelli T., Galasso I. (2010). Phenological growth stages of *Camelina sativa* according to the extended BBCH scale. Ann. Appl. Biol..

[5] Martinez S., Alvarez S., Capuano A., Delgado M.D.M. (2020). Environmental performance of animal feed production from *Camelina sativa* (L.) Crantz: Influence of crop management practices under Mediterranean conditions. Agric. Syst..

[6] Zubr J. (2003). Qualitative variation of *Camelina sativa* seed from different locations. Ind. Crops Prod..

[7] Zubr J. (2009). Unique dietary oil from *Camelina sativa* seed. Agrafood Ind. Hi-Tech.

[8] Bonjean A., Le Goffic F. (1998). *Camelina sativa* (L.) Crantz: Une opportunité pour l’agriculture et l’industrie européennes. OCL Oléagineux Corps Gras Lipides.

[9] Waraich E.A., Ahmed Z., Ahmad R., Ashraf M.Y., Naeem M.S., Rengel Z. (2013). Camelina sativa, a climate proof crop, has high nutritive value and multiple-uses: A review. Austr. J. Crop Sci..

[10] Singh S.K., Rajpurohit B., Singha P. (2021). Camelina (*Camelina sativa*) Seed. Oilseeds: Health Attributes and Food Applications.

[11] Ni Eidhin D., Burke J., O’Beirne D. (2003). Oxidative Stability of ω3-rich Camelina Oil and Camelina Oil-based Spread Compared with Plant and Fish Oils and Sunflower Spread. J. Food Sci..

[12] Tavarini S., De Leo M., Matteo R., Lazzeri L., Braca A., Angelini L. (2021). Flaxseed and Camelina Meals as Potential Sources of Health-Beneficial Compounds. Plants.

[13] Rokka T., Alén K., Valaja J., Ryhänen E.-L. (2002). The effect of a *Camelina sativa* enriched diet on the composition and sensory quality of hen eggs. Food Res. Int..

[14] Ryhänen E.-L., Perttilä S., Tupasela T., Valaja J., Eriksson C., Larkka K. (2007). Effect of*Camelina sativa* expeller cake on performance and meat quality of broilers. J. Sci. Food Agric..

[15] Flachowsky G., Langbein T., Böhme H., Schneider A., Aulrich K. (1997). Effect of false flax expeller combined with short-term vitamin E supplementation in pig feeding on the fatty acid pattern, vitamin E concentration and oxidative stability of various tissues. J. Anim. Physiol. Anim. Nutr..

[16] Peiretti P.G., Mussa P., Prola L., Meineri G. (2007). Use of different levels of false flax *(Camelina sativa* L.) seed in diets for fattening rabbits. Livest. Sci..

[17] Ponnampalam E., Butler K., Muir S., Plozza T., Kerr M., Brown W., Jacobs J., Knight M. (2021). Lipid Oxidation and Colour Stability of Lamb and Yearling Meat (Muscle *Longissimus Lumborum*) from SheepSupplemented with Camelina-Based Diets after Short-, Medium-, and Long-Term Storage. Antioxidants.

[18] Matthäus B., Zubr J. (2000). Variability of specific components in *Camelina sativa* oilseed cakes. Ind. Crops Prod..

[19] Matthäus B., Angelini L.G. (2005). Anti-nutritive constituents in oilseed crops from Italy. Ind. Crops Prod..

[20] Czerniawski P., Piasecka A., Bednarek P. (2021). Evolutionary changes in the glucosinolate biosynthetic capacity in species representing Capsella, Camelina and Neslia genera. Phytochemistry.

[21] Colombini S., Broderick G.A., Galasso I., Martinelli T., Rapetti L., Russo R., Reggiani R. (2014). Evaluation of *Camelina sativa* (L.) Crantz meal as an alternative protein source in ruminant rations. J. Sci. Food Agric..

[22] Hurtaud C., Peyraud J.L. (2007). Effects of Feeding Camelina (Seeds or Meal) on Milk Fatty Acid Composition and Butter Spreadability. J. Dairy Sci..

[23] Pikul J., Wójtowski J., Danków R., Teichert J., Czyżak-Runowska G., Cais-Sokolińska D., Cieślak A., Szumacher-Strabel M., Bagnicka E. (2014). The effect of false flax (*Camelina sativa*) cake dietary supplementation in dairy goats on fatty acid profile of kefir. Small Rumin. Res..

[24] Szumacher-Strabel M., Cieślak A., Zmora P., Pers-Kamczyc E., Bielińska S., Stanisz M., Wójtowski J. (2011). *Camelina sativa* cake improved unsaturated fatty acids in ewe’s milk. J. Sci. Food Agric..

[25] Jambrenghi A.C., Colonna M.A., Giannico F., Cappiello G., Vonghia G. (2007). Effect of goat production systems on meat quality and Conjugated Linoleic Acid (CLA) content in suckling kids. Ital. J. Anim. Sci..

[26] Jambrenghi A.C., Colonna M.A., Giannico F., Coluccia A., Crocco D., Vonghia G. (2009). Meat quality in suckling kids reared by different production systems. Progr. Nutr..

[27] Morsy T.A., Kholif S.M., Kholif A.E., Matloup O.H., Salem A.Z.M., Abu Elella A. (2015). Influence of Sunflower Whole Seeds or Oil on Ruminal Fermentation, Milk Production, Composition, and Fatty Acid Profile in Lactating Goats. Asian Australas. J. Anim. Sci..

[28] Morsy T.A., Mohamed A.G., Zayan A.F., Sayed A.F. (2015). Physicochemical and Sensory Characteristics of Processed Cheese Manufactured from the Milk of Goats Supplemented with Sunflower Seed or Sunflower Oil. Int. J. Dairy Sci..

[29] del Prato O.S., del Prato O.S. (2001). Caciotte. Trattato di Tecnologia Casearia.

[30] Di Trana A., Sepe L., Di Gregorio P., Di Napoli M.A., Giorgio D., Caputo A.R., Claps S. (2015). The role of local sheep and goat breeds and their products as a tool for sustainability and safeguard of the Mediterranean environment. The Sustainability of Agro-Food and Natural Resource Systems in the Mediterranean Basin.

[31] Cosentino C., Colonna M., Musto M., Dimotta A., Freschi P., Tarricone S., Ragni M., Paolino R. (2021). Effects of dietary supplementation with extruded linseed and oregano in autochthonous goat breeds on the fatty acid profile of milk and quality of Padraccio cheese. J. Dairy Sci..

[32] Jambrenghi A.C., Colonna M., Giannico F., Favia R., Minuti F., Scafizzari M., Vonghia G. (2005). Dietary supplementation of garlic and rosemary: Effects on colour stability and lipid oxidation in lamb meat. Ital. J. Anim. Sci..

[33] Ragni M., Tufarelli V., Pinto F., Giannico F., Laudadio V., Vicenti A., Colonna M.A. (2015). Effect of Dietary Safflower Cake (*Carthamus tinctorius* L.) on growth performances carcass composition and meat quality traits in Garganica breed kids. Pak. J. Zool..

[34] Facciolongo A., Lestingi A., Colonna M., Nicastro F., De Marzo D., Toteda F. (2018). Effect of diet lipid source (linseed vs. soybean) and gender on performance, meat quality and intramuscular fatty acid composition in fattening lambs. Small Rumin. Res..

[35] Rotondi P., Colonna M.A., Marsico G., Giannico F., Ragni M., Facciolongo A.M. (2018). Dietary Supplementation with Oregano and Linseed in Garganica Suckling Kids: Effects on Growth Performances and Meat Quality. Pak. J. Zoöl..

[36] Lestingi A., Colonna M., Marsico G., Tarricone S., Facciolongo A. (2019). Effects of legume seeds and processing treatment on growth, carcass traits and blood constituents of fattening lambs. S. Afr. J. Anim. Sci..

[37] Colonna M., Rotondi P., Selvaggi M., Jambrenghi A.C., Ragni M., Tarricone S. (2020). Sustainable Rearing for Kid Meat Production in Southern Italy Marginal Areas: A Comparison among Three Genotypes. Sustainability.

[38] Selvaggi M., Dario C. (2015). Genetic analysis of milk production traits in Jonica goats. Small Rumin. Res..

[39] Tedone L., De Lisi A., Bruno C., De Mastro G. Aspetti produttivi e qualitativi sulla Camelina coltivata in ambiente meridionale (Production and qualitative aspects of Camelina grown in a Southern environment). Proceedings of the IV Convegno Nazionale Piante Mediterranee.

[40] Menke K.H., Raab L., Salewski A., Steingass H., Fritz D., Schneider W. (1979). The estimation of the digestibility and metabolizable energy content of ruminant feedingstuffs from the gas production when they are incubated with rumen liquor in vitro. J. Agric. Sci..

[41] Getachew G., DePeters E., Robinson P., Taylor S. (2001). In vitro rumen fermentation and gas production: Influence of yellow grease, tallow, corn oil and their potassium soaps. Anim. Feed. Sci. Technol..

[42] AOAC (2004). Official Methods of Analysis.

[43] Folch J., Lees M., Sloane-Stanley G.H. (1957). A simple method for the isolation and purification of total lipides from animal tissues. J. Biol. Chem..

[44] Christie W.W. (1982). Lipid analysis-isolation, separation, identification and structural analysis of lipids. Lipid Analysis.

[45] Caponio F., Alloggio V., Pallavicini C. (1998). Modification of goat milk fat triglycerides by immobilized lipase. Lipid/Fett.

[46] Ulbricht T.L.V., Southgate D.A.T. (1991). Coronary heart disease: Seven dietary factors. Lancet.

[47] Osmari E., Cecato U., Macedo F., Souza N. (2011). Nutritional quality indices of milk fat from goats on diets supplemented with different roughages. Small Rumin. Res..

[48] Ivanova A., Hadzhinikolova L. (2015). Evaluation of nutritional quality of common carp (*Cyprinus carpio* L.) lipids through fatty acid ratios and lipid indices. Bulg. J. Agric. Sci..

[49] ASPA (1991). Metodologie Relative alla Macellazione Degli Animali di Interesse Zootecnico e alla Valutazione e Dissezione Della loro Carcassa (Procedures for Slaughter of Livestock Animals and Evaluation and Dissection of Their Carcasses).

[50] Larraín R., Schaefer D., Reed J. (2008). Use of digital images to estimate CIE color coordinates of beef. Food Res. Int..

[51] Todaro M., Palmeri M., Settanni L., Scatassa M.L., Mazza F., Bonanno A., Di Grigoli A. (2017). Effect of refrigerated storage on microbiological, chemical and sensory characteristics of a ewes’ raw milk stretched cheese. Food Packag. Shelf Life.

[52] Rolinec M., Bíro D., Šimko M., Juráček M., Gálik B., Ondrejáková K., Hanušovský O. (2018). The effect of feeding change on nutrients and minerals composition of goat’s milk. J. Central Eur. Agric..

[53] Kováčová M., Výrostková J., Dudriková E., Zigo F., Semjon B., Regecová I. (2021). Assessment of Quality and Safety of Farm Level Produced Cheeses from Sheep and Goat Milk. Appl. Sci..

[54] Shuvarikov A.S., Pastukh O.N., Zhukova E.V., Zheltova O.A. (2021). The quality of milk of goats of Saanen, Alpine and Nubian breeds. IOP Conf. Ser. Earth Environ. Sci..

[55] Dambrosio A., Ioanna F., Storelli M.M., Castiglia D., Giannico F., Colonna M.A., De Rosa M., Quaglia N.C. (2018). Microbiological quality and safety of cheeses belonging to “Traditional Agri-Food Products” (T.A.P.) produced in Southern Italy. J. Food Saf..

[56] Kim J.H., Kim Y., Kim Y.J., Park Y. (2016). Conjugated Linoleic Acid: Potential Health Benefits as a Functional Food Ingredient. Annu. Rev. Food Sci. Technol..

[57] Hartigh L.J.D. (2019). Conjugated Linoleic Acid Effects on Cancer, Obesity, and Atherosclerosis: A Review of Pre-Clinical and Human Trials with Current Perspectives. Nutrients.

[58] Cossignani L., Giua L., Urbani E., Simonetti M.S., Blasi F. (2014). Fatty acid composition and CLA content in goat milk and cheese samples from Umbrian market. Eur. Food Res. Technol..

[59] Bessa R., Santos-Silva J., Ribeiro J., Portugal A. (2000). Reticulo-rumen biohydrogenation and the enrichment of ruminant edible products with linoleic acid conjugated isomers. Livest. Prod. Sci..

[60] Zunong M., Hanada M., Aibibula Y., Okamoto M., Tanaka K. (2008). Variations in Conjugated Linoleic Acid Concentrations in Cows Milk, Depending on Feeding Systems in Different Seasons. Asian Australas. J. Anim. Sci..

[61] Pajor F., Galló O., Steiber O., Tasi J., Póti P. (2009). The effect of grazing on the composition of conjugated linoleic acid isomers and other fatty acids of milk and cheese in goats. J. Anim. Feed. Sci..

[62] Wood J.D., Richardson R.I., Nute G.R., Fisher A.V., Campo M., Kasapidou E., Sheard F.P., Enser M. (2004). Effects of fatty acids on meat quality: A review. Meat Sci..

[63] Dauber C., Carreras T., Britos A., Carro S., Cajarville C., Gámbaro A., Jorcin S., López T., Vieitez I. (2021). Elaboration of goat cheese with increased content of conjugated linoleic acid and transvaccenic acid: Fat, sensory and textural profile. Small Rumin. Res..

[64] Calder P.C. (2012). Mechanisms of Action of (*n*-3) Fatty Acids. J. Nutr..

[65] Silva M.D.F.S., Bessa R. (2002). Effect of genotype, feeding system and slaughter weight on the quality of light lambs. Livest. Prod. Sci..

[66] Paszczyk B., Łuczyńska J. (2020). The Comparison of Fatty Acid Composition and Lipid Quality Indices in Hard Cow, Sheep, and Goat Cheeses. Foods.

[67] Innosa D., Ianni A., Faccia M., Martino C., Grotta L., Saletti M.A., Pomilio F., Martino G. (2020). Physical, Nutritional, and Sensory Properties of Cheese Obtained from Goats Fed a Dietary Supplementation with Olive Leaves. Animals.

[68] Martin B., Priolo A., Valvo M.A., Micol D., Coulon J.B., Molina Alcaide E., Ben Salem H., Biala K., Morand-Fehr P. (2005). Effects of grass feeding on milk, cheese and meat sensory prop-erties. Sustainable Grazing, Nutritional Utilization and Quality of Sheep and Goat Products.

[69] Marilley L. (2004). Flavours of cheese products: Metabolic pathways, analytical tools and identification of producing strains. Int. J. Food Microbiol..

